# The prevalence of urethral and rectal *Mycoplasma genitalium* among men who have sex with men in China, a cross-sectional study

**DOI:** 10.1186/1471-2458-14-195

**Published:** 2014-02-21

**Authors:** Bing-jie Zheng, Yue-ping Yin, Yan Han, Mei-qin Shi, Ning Jiang, Zhi Xiang, Rui-xing Yu, Guo-yi Zhang, Xiang-sheng Chen

**Affiliations:** 1 National Center for STD Control, Chinese Academy of Medical Science & Peking Union Medical College Institute of Dermatology, Nanjing, China; 2 Reference Laboratory, 12 Jiangwangmiao Street, Nanjing 210042, China

**Keywords:** *Mycoplasma genitalium*, Prevalence, MSM, China

## Abstract

**Background:**

Although *Mycoplasma genitalium* (MG) is a common sexually transmitted infection (STI), very little information regarding the prevalence of MG among MSM (men who have sex with men) is available in China. The objective of this study was to determine the prevalence of MG among MSM in the city of Shenzhen, Guangdong Province, China, and to identify the potential risk factors associated with MG infection in this population.

**Methods:**

Between January and May 2010, a total of 409 MSM were recruited in Shenzhen, Guangdong Province, China. An anonymous questionnaire was used to collect information regarding their sociological and sexual behaviors. In addition, their first-void urine (FVU) samples and rectal swabs were collected for PCR-based MG testing.

**Results:**

Among the 406 FVU and 405 rectal swab samples were collected from 409 MSM, the overall MG prevalence was 8.1% (33/406, 95% CI 5.7%-10.6%), with a FVU positivity of 3.4% (95% CI 1.7%-5.4%) and a rectal positivity of 5.4% (95% CI 3.5%-7.7%). Using both univariate and multivariable logistic regression analyses, urethral MG infection was significantly associated with having more heterosexual behaviors (AOR 7.16, 95% CI 1.89-27.13), and with having unprotected anal intercourse in the past six months (AOR 4.80, 95% CI 1.40-16.47). Rectal MG infection was significantly associated with HIV infection based on univariate logistic regression analysis (OR = 4.49, 95% CI 1.18-17.12).

**Conclusions:**

In this study, we investigated the prevalence of MG infection in the population of interest, as determined from both urethral and rectal specimen. We showed that MG was more prevalent in MSM who had bisexual behaviors compared to those who engaged only in homosexual behaviors. Further work is needed to establish the mode of MG transmission and to identify its role in HIV transmission. Meanwhile, more attention should be paid to MG infection among MSMs, and especially bisexual MSMs, which might have critical implications for effective HIV/STD control in China.

## Background

Accumulating evidence supports that *Mycoplasma genitalium* (MG) is a common sexually transmitted infection (STI) pathogen that can cause non-gonococcal urethritis (NGU) in men [1-3] and pelvic inflammatory disease (PID) in women [4,5]. In addition, the positive association between MG and human immunodeficiency virus (HIV) has been identified in several studies [6-8]. However, there have been limited number of epidemiological studies on MG infection worldwide and data on the prevalence of this pathogen among men who have sex with men (MSM) are especially scarce. MSM is a critical population because it plays a significant role in the spread of HIV and other sexually transmitted diseases (STD) [9]. Recent studies revealed an increasing trend of HIV/STD prevalence in Chinese MSM [10]. However, to date, there have been no reports on the prevalence of MG in this population. As part of a large cross-sectional research effort aimed at investigating the epidemiology of MG infection among high risk populations in China, this study has two objectives: (i) to determine the prevalence of urethral and rectal MG in MSM in Shenzhen, Guangdong Province, China, and (ii) to identify the potential risk factors for MG infection.

## Methods

### Participants

Between January and May 2010, a total of 409 men older than 18 years old who reported having oral sex or anal sex with men within the past year were recruited for this study. Participants were recruited from a MSM voluntary counseling and testing clinic in Shenzhen, China. This clinic has been operated by a non-governmental organization (NGO) and the local center for disease control (CDC). Those who participated in high-risk behaviors (e.g. MSMs) were generally willing to undergo free counseling and HIV/STD testing at this clinic. Meantime, a free and confidential physical exam was provided if the patient desired. After informed consent was obtained, each participant was asked to fill out an anonymous questionnaire containing questions regarding socio-demographic characteristics, sexual behaviors and STD history information in a private room on his own. After completing the questionnaire, all participants accepted a routine physical examination by a trained doctor according to the standard protocol. Approximately 10-15 ml of FVU samples were collected by the participants themselves, and rectal swabs were collected by trained nurses. The specimens were kept at -20°C until sample collection from all participants was completed. The specimens were then sent to the National Center for STD Control in Nanjing, China on dry ice by a cold-chain transportation. Those who declined to provide either urine or rectal specimens were included in the analysis as unpaired data. The protocols for this study were reviewed and approved by the Medical Ethics Committee at the Institute of Dermatology of the Chinese Academy of Medical Sciences. This study was nested within a multisite cross-sectional screening program investigating the prevalence of STIs and HIV infection among MSMs [11]. Therefore, test results regarding the participants’ HIV positivity were also available to us. We took advantage of these data to analyze the association between MG and HIV infections.

### Laboratory test

After arrival in the laboratory of National Center for STD Control in Nanjing, China, rectal swab specimens were eluted with 500-μl normal saline. Each urine specimen and rectal swab eluent was mixed thoroughly, distributed into aliquots and stored at -70°C until tested for MG. Genomic DNA was extracted from the specimens by QIAmp DNA Mini Kit (Qiagen GmbH, Germany) according to the manufacturer’s protocol. MG DNA was detected using a previously published real time PCR method that targets the *MgPa* adhesion gene [12,13].

### Data analysis

Data were independently entered into a computer database using EpiData software (EpiData Association, Denmark) by two research assistants, and any discrepancies were resolved prior to analysis. Statistical analyses were performed using SPSS for Windows 13 (SPSS, Inc, Chicago, IL, USA). Comparisons of categorical variables were conducted using a chi square test. Univariate analyses were performed to examine the relationships between each variable and MG infection. Only variables that were significant in the univariate analyses with P values < 0.10 were included in multivariable logistic regression models for selecting independent risk factors. Odds ratios (ORs) and their corresponding 95% confidence intervals (CIs) were calculated.

## Results

Between January and May 2010, a total of 409 MSMs were recruited in this study; of them, three declined to provide FVU and four others declined to provide rectal specimens. Thus, 406 urine samples and 405 rectal swabs were collected for detection of MG.

### Demographic and behavioral characteristics

The mean age of MSM participants was 30.5 years old (ranging 18-64 years), and more than half of them were younger than 30 years old. The majority of participants were of Han ethnicity (95.6%, 391/409); 64.5% (264/409) received senior high school or lower education; 75.1% (307/409) were single, divorced, or widowed; 23.7% (97/409) were permanent residents of Guangdong province, China; 45.5% (186/409) were living alone.

Our results showed that 34.3% (134/390) of participants identified themselves as homosexual men and 61.5% (240/390) of participants reported having sexual intercourse only with men or more with men than with women. Overall, 93 (22.9%) of MSMs engaged in commercial sexual behaviors and 96.7% (90/93) of them used condoms during the most recent commercial sex encounters. 386 MSMs reported having anal sex in the past 6 months, of which 275 did not use condoms. Additionally, 106 (26.1%) participants reported having sex with women during the past six months, and 23 (5.6%) reported using illegal substances in the past year.

### MG test results and risk factors

Of the total 406 FVU and 405 rectal samples processed, 14 (3.4%, 95% CI 1.7%-5.4%) FVU samples and 22 (5.4%, 95% CI 3.5%-7.7%) rectal samples were positive for MG DNA by real-time PCR method. Three pairs of urine and rectal specimens were co-positive for MG, giving an overall MG prevalence of 8.1% (33/406, 95% CI 5.7%-10.6%).

Analyses of MG test results from the urethral samples suggest that attendees who engaged in predominantly heterosexual behaviors were nearly five times more likely to be infected with urethral MG than those who were predominantly homosexual (OR 4.56, 95% CI 1.31-15.90, P = 0.017). In addition, having unprotected anal intercourse in the past six months was significantly associated with positive urethral MG (OR 3.53, 95% CI 1.19-10.41, P = 0.023). Multivariate analyses revealed that urethral MG infection was significantly associated with having more heterosexual behaviors (AOR 7.16, 95% CI 1.89-27.13, P = 0.004) and with unprotected anal intercourse (AOR 4.80, 95% CI 1.40-16.47, P = 0.013) (Table 1).

**Table 1:** Risk factors associated with urethra/rectum MG infection

**Characteristic**	**Percentage with characteristic**	**MG infection number (%)**	**Crude odds ratio (95% CI), P value**	**Adjusted odds ratio (95% CI), P value**
Urethral MG				
Sexual partners				
Only men or predominantly men	346 (89.4)	8 (2.3)	1	
Almost equally or predominantly women	41 (10.6)	4 (9.8)	4.57 (1.31–15.90), 0.017*	7.16 (1.89–27.13), 0.004
Unprotected anal intercourse in the past 6 mo				
No	273 (71.5)	6 (2.2)	1	
Yes	109 (28.5)	8 (7.3)	3.53 (1.19–10.41), 0.023*	4.80 (1.40–16.47), 0.013
Rectal MG				
HIV Infection				
Yes	16 (4.0)	3 (18.8)	4.49 (1.18–17.12), 0.028	
No	389 (96.0)	19 (5.1)	1	

*included in multivariable analysis.

Analyses of MG test results from the rectal samples suggest a significant association between rectal MG and HIV infection, with an OR of 4.49 (95% CI 1.18-17.12, P = 0.028) (Table 1).

We did not find significant associations between overall MG infections and any demographic factors, urethral or rectal symptoms, self-identified sexual orientation, and commercial sex behaviors. No significant correlation was observed between overall MG and HIV infections (OR 2.79, 95% CI 0.75-10.86, P = 0.124) although the prevalence of MG was higher in HIV-positive attendees (18.7%) than in HIV-negative attendees (7.6%). In addition, of those three participants with dual MG positivity in FVU and rectal specimens, one was co-infected with HIV (Table 2).

**Table 2:** Risk factors associated with overall MG infection

**Characteristic**	**Number (%) with characteristic**	**MG infection number (%)**	**Crude odds ratio (95% CI)**	**P value**
N = 409				
Age in years				
≤30	233 (57.0)	20 (8.6)	1.18 (0.57–2.44)	0.660
>30	176 (43.0)	13 (7.4)	1	
Ethnicity				
Han	391 (95.6)	32 (8.2)	1.52 (0.20–11.76)	0.691
Other	18 (4.4)	1 (5.6)	1	
Education				
Senior high school or lower	264 (64.5)	24 (9.1)	1.51 (0.68–3.34)	0.308
Junior college or higher	145 (35.5)	9 (6.2)	1	
Marital status				
Unmarried	307 (75.1)	26 (8.5)	1.26 (0.53–2.99)	0.606
Married	102 (24.9)	7 (6.9)	1	
Residential status				
Guangdong Province	97 (23.7)	5 (5.2)	1	
Other Province	312 (76.3)	28 (9.0)	1.81 (0.68–4.83)	0.234
Cohabiting status				
Living alone	186 (45.5)	18 (9.7)	1.09 (0.45–2.60)	0.855
Living with spouse or family	73 (17.8)	2 (2.7)	0.29 (0.06–1.39)	0.120
Living with men	61 (14.9)	5 (8.2)	0.90 (0.28–2.91)	0.866
Living with women or others	89 (21.8)	8 (9.0)	1	
Presenting STI symptoms in the past year				
Yes	35 (8.6)	5 (14.3)	2.06 (0.74–5.72)	0.166
No	374 (91.4)	28 (7.5)	1	
Sexual orientation				
Homosexual only	134 (34.4)	10 (7.5)	0.57 (0.11–2.84)	0.488
Bisexual (homosexual predominance)	240 (61.5)	18 (7.6)	0.57 (0.12–2.69)	0.476
Bisexual (heterosexual predominance)	16 (4.1)	2 (12.5)	1	
Sexual partners				
Only men or predominantly men	349 (89.4)	26 (7.4)	1	
Almost equally or predominantly women	41 (10.5)	4 (9.8)	1.34 (0.44–4.06)	0.601
Commercial sex work				
Sometimes	93 (22.9)	5 (5.4)	0.58 (0.22–1.54)	0.274
Never	313 (77.1)	28 (8.9)	1	
Anal intercourse in the past 6 mo				
Yes	386 (95.1)	32 (8.3)	1.72 (0.22–13.25)	0.604
No	20 (4.9)	1 (5.0)	1	
Unprotected anal intercourse in past 6 mo				
No	275 (71.4)	20 (7.3)	1	
Yes	110 (28.5)	12 (10.9)	1.56 (0.74–3.31)	0.246
Vaginal sex in the past 6 mo				
Yes	106 (26.1)	5 (4.7)	0.48 (0.18–1.28)	0.141
No	299 (73.8)	28 (9.4)	1	
Place for seeking sexual partners				
Non-internet	177 (43.3)	10 (5.6)	0.53 (0.25–1.15)	0.110
Internet	228 (55.7)	23 (10.1)	1	
Illegal substance use in the past year				
Yes	23 (5.7)	1 (4.3)	0.50 (0.07–3.80)	0.496
No	381 (93.2)	32 (8.4)	1	
HIV Infection				
Yes	16 (3.9)	3 (18.7)	2.79 (0.75–10.34)	0.124
No	393 (96.7)	30 (7.6)	1	

## Discussion

There are a few reports on the prevalence of MG infections among MSM populations in both urine and rectal specimens [14-16]. Among MSMs in London, the overall MG prevalence was 6.6% with 2.7% positive in FVU and 4.6% positive in rectal specimens [15]. A cross-sectional study of 521 MSMs in Melbourne reported these rates to be 2.1%, 0.6%, and 1.6%, respectively [14], and Reinton *et al*. reported 5.1%, 1.7% and 4.5%, respectively, in the studied population of Norway [16]. Our present study found that MSMs in Shenzhen, China had an overall MG infection rate of 8.1%, with 3.4% positive in FVU and 5.4% positive in rectal specimens. Compared to the above-mentioned studies, this study showed higher prevalence of overall MG infection as well as in both anatomic sites. Given that MG prevalence of male STD attendees and female sex workers (FSWs) in the studied area were higher than in other countries [13], the high prevalence in this study may indicate a MG epidemic in this region. In addition, regardless of differences in MG detection method and MG prevalence in different areas, all studies have found that MG was more likely to be detected in the rectal specimens than in FVU in the MSM population, indicating that anorectal mucosa might be easily infected with MG in the population who had anal sex. Interestingly, the same phenomenon has been frequently reported in studies on the epidemiology of CT infection among MSMs, with 4.0%-7.9% positive in rectal specimens and 1.5%-5.2% positive in urethral specimens [14,17-20]. These studies also found that rectal CT infections were more asymptomatic than urethral infections. However, it is not clear whether rectal MG infection is also asymptomatic compared to urethral infection. Regardless of whether rectal MG infection is asymptomatic, or whether anorectal mucosa is easily infected*,* it is becoming clear that the rectum is a core anatomic site for both MG and CT infections in the MSM population. Currently, the Centers for Disease Control (CDC) guidelines recommend that STD clinics offer CT rectal screening or diagnostic testing, but have not given a similar suggestion for MG, and few STD clinics perform routine screening of rectal or urethral MG for urethritis/proctitis patients or asymptomatic patients. Considering the higher prevalence of rectal MG infection, identifying the populations with rectal infection is a key component of successful MG surveillance and control among MSMs.

In the present study, MG was more common among HIV-infected individuals (18.7%) as compared to those without HIV infection (7.6%), but the difference was not statistically significant. This is consistent with previous studies among male NGU patients, which also showed insignificant correlation between these two pathogens [21,22]. Notably, we found that the risk of rectal MG infection was significantly higher (5 times) in MSMs who had an HIV infection compared to those who did not have an HIV infection. However, the risk of urethral MG infection only doubled with HIV infection and the difference was not statistically significant. This discrepancy in MG and HIV association might in part reflect the sexual behaviors of these high risk populations: compared with the general population, HIV-positive men are more likely to change sexual partners frequently and more likely to have unprotected anal sex [23,24]. On the other hand, immunosuppression and T-cell dysfunction caused by HIV could increase the susceptibility to MG [25]. Savio *et al*. reported that MG was more commonly detected among acquired immune deficiency syndrome (AIDS) patients compared to those asymptomatic, HIV-positive individuals [26]. Meanwhile, the potential acute or chronic inflammation of rectal mucosa caused by MG may also play a role in HIV infection. However, to date, it is unclear whether the increased risk of HIV infection is due to MG infection. Previous longitudinal studies reported over two-fold higher risk of HIV acquisition by women infected with MG than by those without MG, and 8.7% of HIV-1 acquisition were attributable to prior MG infection [27]. Further research is needed to establish the causal relationship between MG and HIV infections.

At present, the MSM population has become one of the high-risk groups for HIV/STD infection because of their active sexual activities without protection [9]. According to a systematic review and meta-analysis on HIV incidence among MSMs in China, about 33% of new HIV cases in 2009 occurred in MSMs who account for only 2-4% of the Chinese adult male population [28]. More attention has been paid to this population in preventing HIV/STD transmission. However, the main focus has been on preventing syphilis and other STIs, while little effort has been devoted to MG detection and prevention. Our results showed an association between HIV and MG infections, which might highlight the importance of testing of MSMs for MG, especially in the rectum.

Notably, we have found that MSMs who engaged in predominantly heterosexual behaviors were more likely to be infected with urethral MG than those who were predominantly homosexual. This result is similar to findings from previous studies among male STD patients [29,30]. This might be attributed to more unprotected vaginal and anal intercourses in this population than in MSMs who engaged in exclusively homosexual behaviors. Several studies have reported that urethral MG infection was associated with unprotected vaginal sex [2,30]. The prevalence of MG infection in FSWs and heterosexual men, ranging from 12% to 26% [17,31-33], was higher than that in MSMs, from 4.5% to 26% [34]. Furthermore, MG infection can persist for a long time in the female genital tract [33,31,35]. Thus, it is plausible to conclude that vaginal sexual intercourse is a major route of transmission for MG. Perhaps, MSMs with bisexual behaviors is a sub-population with higher risk for MG infection. It was previously reported that more than one-third of MSMs in China were married to women, and more than 70% of heterosexual behaviors were unprotected [36]. Therefore, strengthening the screening of MG among MSM, especially those who had bisexual behaviors, is an important strategy to control this pathogen. In addition, identifying the hidden bisexual MSM with MG infection is helpful for successful HIV/STD control.

The limitations of present study are that reporting bias might exist. Compared with web-based or computer-based questionnaire, the present information-collection method may have less controllability and may be susceptible to misreporting. Besides, we did not distinguish between “insertive” and “receptive” anal intercourse among the studied population. Finally, we did not collect data regarding current symptoms and timeframe of sexual partner.

## Conclusions

In this study, we provided MG prevalence at both urethral and rectal sites among the MSM population in Shenzhen, China. We showed that MG was more prevalent in MSMs who had bisexual behaviors compared to those who engaged mainly in homosexual behaviors. It should be noted that there is currently little attention given to MG infection in the National HIV/STD Prevention and Surveillance Guidelines and by health-care providers in China. Further work is needed to establish the mode of MG transmission and to identify its role in HIV transmission. Considering that the MSMs sexually interact with other populations (such as FSWs and the general female population) and therefore acting as a bridge for HIV/STD transmission, paying more attention to MG infection among MSMs, and especially bisexual MSMs, might have important implications for HIV/STD control.

## Competing interests

The authors declare that they have no competing interests.

## Authors’ contributions

BJZ, YPY and XSC designed the study and wrote the initial manuscript. NJ and XSC assisted the field work and data collection. YH, MQS, ZX and RXY did the laboratory test. BJZ and YH did the data analysis. XSC and GYZ edited the manuscript and completed the final revisions. All authors read and approved the final manuscript.

## Pre-publication history

The pre-publication history for this paper can be accessed here:

http://www.biomedcentral.com/1471-2458/14/195/prepub
